# Exploring Generational Differences of British Ethnic Minorities in Smoking Behavior, Frequency of Alcohol Consumption, and Dietary Style

**DOI:** 10.3390/ijerph16122241

**Published:** 2019-06-25

**Authors:** Senhu Wang, Shuanglong Li

**Affiliations:** 1 Centre for Business Research, University of Cambridge, 11-12 Trumpington Street, Cambridge CB2 IAG, UK; sw768@cam.ac.uk; 2 Department of Sociology, School of Public Administration, Guangzhou University, Guangzhou 510006, China

**Keywords:** ethnic minority immigrants, migration generation, smoking, alcohol consumption, dietary style, health behavior, ethnic identity

## Abstract

*Background:* This article explores ethnic minority generational differences in smoking behavior, frequency of alcohol consumption, and dietary style in Britain, and whether these differences can be explained by generational differences in socioeconomic status and ethnic identity. *Method:* Multivariate analyses using wave 2 (2010–2012) and wave 5 (2013–2015) of the United Kingdom Household Longitudinal Study on smoking behavior, frequency of alcohol consumption, and dietary style from 59,189 White British, 1690 Indians, 960 Pakistanis, 555 Bangladeshis, 1060 Black Caribbeans, and 1059 Black Africans, adjusted for demographic characteristics, socioeconomic status and ethnic identity. *Results:* While we find little evidence for generational differences in dietary style, second-generation Indians, Pakistanis, and Black Caribbeans have a significantly higher probability of smoking than the first-generation, and all second-generation minorities are significantly more likely to consume alcohol than their first-generation counterparts. Such generational differences in alcohol consumption are partly explained by second-generation minorities’ weakened ethnic identity and higher socioeconomic status. *Conclusions:* This study facilitates a better understanding of minority generational differences in health behaviors and the role of socioeconomic status and ethnic identity, highlighting the need for future policy interventions to target certain second-generation ethnic minorities who have adopted certain host society unhealthy lifestyles.

## 1. Introduction

Previous research in Britain shows that newly arrived immigrants tend to have better health than their non-migrant and host society counterparts due to self-selection effects, but their health advantages tend to diminish over time and generations. This is known as the migrant health paradox [1,2]. It is argued that an essential reason for the generational deterioration of health could be generational changes in health behaviors and lifestyles, highlighting the importance of understanding ethnic minorities’ generational transition in health behaviors [3].

In Britain, relatively large immigrant groups (e.g., Indians, Pakistanis, Bangladeshis, Black Caribbeans, and Black Africans) mainly come from South Asian, the Caribbean, and Sub-Saharan African regions [4]. In these areas, local cultural and religious norms often prohibit or discourage certain detrimental health behaviors such as tobacco and alcohol use, emphasize asceticism and sacredness of the body, and encourage some healthy lifestyles such as vegetarian diets [2,5,6]. In addition, the cultures of these regions tend to emphasize obedience to parents and authority, family honor, and interdependence of family members, which could in turn prevent adolescents from socializing deviant and detrimental health behaviors [7]. In contrast, the cultures of many migrant-receiving countries such as Britain often emphasize individualism, self-expression, and self-actualization, and are thus more tolerant towards different lifestyles including tobacco smoking, alcohol consumption, and substance use [8]. As first-generation British immigrants grew up and were socialized in their country of origin, their home country cultures could, to some extent, protect them against the host society’s deviant and unhealthy lifestyles [9]. For example, many British first-generation immigrants, especially Pakistanis and Bangladeshis, tend to cluster and concentrate in urban ethnic enclaves. The distinctive norms within cohesive ethnic enclaves can facilitate the socialization of norms and exert social control over behavior in ways that accord with those of the home country, therefore preventing them from adopting deviant and unhealthy lifestyles from the host society [9].

In terms of the second-generation, classical straight-line assimilation theory suggests that the longer immigrants stay in a host country, the more they are integrated or assimilated into a host society, characterized by upward social mobility, reduced ethnic distinctiveness, dispersive residence, mixed marriage, and friendships [10]. Research shows that British second-generation ethnic minorities who were socialized in Britain not only tend to have a weaker ethnic identity and attach less importance to their home country cultures, but also have higher levels of inter-ethnic friendship and marriage rates, and reside in more ethnically mixed areas than first-generation migrants [11,12]. These patterns suggest that, compared to the first-generation, British second-generation ethnic minorities become overall more similar to White British and are likely to accept and adopt host society health behaviors and lifestyles including some unhealthy ones such as tobacco smoking, alcohol consumption, and high-calorie food intake, which could in turn undermine their health [3].

However, segmented assimilation theory challenges the predictions of classical straight-line assimilation theory by arguing that the different resources of immigrants (e.g., human capital, family structure, mode of incorporations, discrimination, and labor market contexts) may intersect to result in different assimilation outcomes [13]. The theory has explicitly argued that some ethnic groups may undergo selective assimilation where they achieve upward social mobility in the host country while maintaining the cultural tradition from their home country [13], suggesting that not all ethnic groups will gradually adopt host society health behaviors and lifestyles over generations. This is especially the case in Britain where a series of multicultural policies (defined as policies promoting cultural diversity and protecting ethnic minorities’ rights) in recent decades (e.g., the Race Relations Act from 1965 to 2000 and the 2010 Equality Act) have been implemented to ensure the rights and entitlements of ethnic minorities, such as the provision of special meals for Muslim students, exemptions from standard British dress code, and funding for minority group organizations [14,15]. These policies are thought to encourage ethnic minorities to live ‘parallel lives’ in segregated communities [16]. According to this view, the high levels of ethnic segregation are likely maintaining ethnic minority healthy lifestyles derived from their home countries and prevent them from adopting some host society unhealthy lifestyles.

Given the different predictions from classical straight-line assimilation theory and segmented assimilation theory, it is crucial to systematically explore British ethnic minorities’ generational changes in health behaviors. To date, most research on ethnic minorities’ generational differences in health behaviors comes from North America and generally supports the argument that second or later-generation ethnic minorities are more likely to smoke, drink alcohol, and eat high-calorie food than their first-generation counterparts [5,6,17]. Given the significant differences in ethnic composition and ethnic policies across countries, the findings from previous research in other countries cannot be easily generalized to Britain. However, in Britain, there have only been a few studies that focused on ethnic minorities’ generational differences in health behaviors, but these studies either solely focused on a small sample [18,19], or only used simple descriptive statistics to compare the generational differences in health behaviors [3].

Thus, this article’s objective is to explore the generational differences in health behaviors and see whether the differences, if any, can be explained by their socioeconomic status (SES) and degree of ethnic identity. If the second-generation minorities are more likely to adopt unhealthy lifestyles than the first-generation, policy interventions to boost healthy lifestyle and ethnic health equity may need to be more targeted at these population groups. However, if health behaviors do not vary over minority generations, scholars and policymakers may need to focus on other factors that may contribute to ethnic minorities’ poor health, such as discrimination, racial harassment, and economic inequalities.

## 2. Methods

### 2.1. Data and Sample

In this study, the data come from wave 2 (2010–2012) and wave 5 (2013–2015) of the United Kingdom Household Longitudinal Study (UKHLS). Launched in 2009, the UKHLS contains high-quality longitudinal data covering a variety of subjects such as family, work, health, and education, and it aims to enable a better understanding of large-scale socioeconomic changes [20]. We used both waves of the UKHLS because they are the only waves that have detailed and consistent measures of respondents’ health behaviors and ethnic identity, and the response rate is 61%. The UKHLS comprises a multistage stratified and clustered general population sample (GPS) of about 40,000 households as well as an ethnic minority boost sample (EMBS) that was designed to yield about 1000 respondents for the five major ethnic minorities in Britain: Indian, Pakistani, Bangladeshi, Black Caribbean, and Black African [20]. Thus, cross-sectional weights provided by the UKHLS are used to adjust for the non-response rate and unequal selection probabilities. The data collected were reviewed and approved by the university institutional review board.

### 2.2. Measures

#### 2.2.1. Ethnicity

Ethnicity is measured by self-identified ethnic classification. Respondents were asked to select the ethnic group to which they thought they belonged from a list of 18 ethnic categories (for more details, see https://www.understandingsociety.ac.uk/). Using this approach, White British are defined as the respondents who identify themselves as ‘White British/English/Scottish/Northern Irish’. Similarly, the five minority groups (i.e., Pakistani, Bangladeshi, Indian, Black African, and Black Caribbean) were identified.

#### 2.2.2. Migration Generation

Previous research [4,21] was followed in order to distinguish further the five minority groups between first (born overseas) and second or later generations (born in or arrived in the UK before the compulsory schooling age, i.e., five). Although it is argued that this ethnic classification may conceal important cultural and religious heterogeneities within ethnic groups [22], this study still uses this classification for consistency and easy comparability with previous research.

#### 2.2.3. Health Behaviors

The dependent variables are three health behavior indicators. Smoking behavior is a binary variable that measures whether respondents currently smoke or not: 1 (Yes) and 0 (No). The frequency of alcohol consumption is an ordinal variable including eight categories: 1 (not at all in last 12 months), 2 (once or twice a year), 3 (once every couple of months), 4 (once or twice a month), 5 (once or twice a week), 6 (three or four days a week), 7 (five or six days a week), and 8 (almost every day). The number of servings of fruits and vegetables eaten per day (ranges from 0 to 26) is a count variable that measures the health level of dietary style.

#### 2.2.4. Covariates

This study also includes three types of covariates: demographic characteristics, SES, and ethnic identity. These factors have been shown to have a significant impact on people’s health behaviors [17].

Demographic characteristics include respondents’ age, gender, and marital status (including three categories: single, married/cohabited, and separated/divorced/widowed).

SES includes two indicators: respondents’ occupational class measured by The National Statistics Socio-economic Classification (including five categories: inactive, unemployed, working class, intermediate class, and salariat class), and logged equivalized household income (adjusted for the number of people in the household).

Ethnic identity is measured by a question asking respondents about the extent to which ethnic background is important to their identity on a four-point ordinal scale: 1 (very important), 2 (fairly important), 3 (not very important), and 4 (not important). It is widely argued that weaker ethnic minority identities are an essential sign of ethnic assimilation [22,23].

### 2.3. Modeling Strategy

This study uses Logistic and Poisson regression models to predict smoking behavior (binary variable) and the number of servings of fruits and vegetables eaten per day (count variable), respectively. As the frequency of alcohol consumption generally follows a normal distribution (skewness = 0.16), it is treated as a continuous variable and analyzed by linear regression models. To explore whether ethnic minority health behaviors gradually converge towards those of White British over generations, we first compared White British to first and second-generation ethnic minorities, respectively, controlling for demographic characteristics. Next, in order to explore the generational differences within ethnic minorities and the role of SES and ethnic identity, we restricted the sample to ethnic minorities and compared the first to the second-generation minorities, while adding demographic characteristics, SES and ethnic identity stepwise into the models.

## 3. Results

### 3.1. Descriptive Statistics

Table 1 reports descriptive statistics by ethnic groups. Overall, all ethnic minority groups are younger and are more likely to reside in urban areas than White British. While Indians, Pakistanis, and Bangladeshis are more likely to be married than White British, and Black Caribbeans and Black Africans are less likely to be married than White British. In addition, all ethnic minorities except Indians have lower SES (occupational class and household income) than White British. In terms of health behaviors, all ethnic minorities are less likely to be current smokers, have lower levels of alcohol consumption, and eat fewer servings of fruits/vegetables per day compared to White British.

To understand the generational changes, Table 2 further reports the descriptive statistics by generational status within each ethnic minority group. Overall, second-generation ethnic minorities are younger, less likely to be married, and have higher levels of occupational class and household income than their first-generation counterparts. In terms of health behaviors, all second-generation ethnic minorities are more likely to smoke, have higher levels of alcohol consumption, and eat fewer servings of fruits/vegetables per day compared to the first-generation. Finally, compared to the first-generation, all second-generation ethnic minority groups are less likely to attach great importance to their ethnic background.

### 3.2. Regression Analysis

#### 3.2.1. Smoking Behavior

After adjusting for demographic characteristics, Model 1 in Table 3 shows that all first- and second-generation minorities except first-generation Bangladeshis and second-generation Black Caribbeans are significantly less likely to smoke than White British. In terms of generational differences, Model 2 show that, after controlling for demographic characteristics, second-generation Indians, Pakistanis, and Black Caribbeans are 10%, 8%, and 21% more likely to smoke than their first-generation counterparts, respectively. However, after adding SES and ethnic identity into the models, the generational differences generally remain similar, suggesting that both factors do not play a crucial role in explaining the generational differences. For robustness of the results, Table A1 in the Appendix repeats the above analysis by comparing ethnic and generational differences with the probability of being ever-smoker vs. never-smoker. Similarly, second-generation Indians, Pakistanis, Black Caribbeans, and Black Africans are 20%, 14%, 28%, and 14% more likely to smoke than their first-generation counterparts, respectively. These patterns remain similar after controlling for SES and ethnic identity.

#### 3.2.2. Frequency of Alcohol Consumption

Model 1 in Table 4 shows that all ethnic minorities, regardless of migration generation, have significantly lower frequencies of alcohol consumption than White British, but the ethnic differences are less pronounced among the second-generation ethnic minorities. Consistent with these findings, Model 2 shows that all second-generation ethnic minorities, especially Indians, Bangladeshis, and Black Caribbeans have significantly higher frequencies of alcohol consumption than their first-generation counterparts. As can be seen from Model 3, after controlling for SES, the generational differences are to some extent attenuated in size for all ethnic groups except Pakistanis. In particular, the generational difference becomes non-significant for Black Africans. Further Sobel–Goodman mediation tests suggest that around 7–11% of generational differences are significantly mediated by SES for these groups (*p* < 0.05). With ethnic identity entered in Model 4, the generational differences remain similar but are to some extent attenuated in size for Bangladeshis. Further Sobel–Goodman mediation tests suggests that around 17% of the generational gap is significantly mediated by an ethnic identity for Bangladeshis (*p* < 0.05).

#### 3.2.3. Dietary Style

Model 1 in Table 5 shows that after controlling for demographic characteristics, all ethnic minorities tend to eat significantly fewer fruits/vegetables than White British. Moreover, the ethnic differences are generally more pronounced among second-generation Indians, Black Caribbeans, and Black Africans. Model 2 shows that holding demographic characteristics constant, second-generation Indians tend to eat significantly fewer fruits/vegetables than their first-generation counterparts, and the generational difference remains similar even after controlling for SES and ethnic identity. In addition to Indians, there are no significant generational differences for other ethnic minority groups. To ensure the robustness of the results, we have recorded the number of servings of fruits/vegetables eaten per day into a binary variable, i.e., whether five or more servings of fruits/vegetables are consumed per day, and conducted logistic regression analysis, as shown in Table A2. Consistent with the results in Table 5, ethnic minorities are on average less likely to eat five or more servings of fruits/vegetables than White British. There is little evidence for generational differences in dietary style for most ethnic minorities.

## 4. Discussion

As the Race Equality Scheme of the Department of Health in Britain states, ‘the NHS increasingly needs to take into account not only cultural and linguistic diversity but also needs to be able to cater for varying lifestyles and faiths’ (The Department of Health, 2005, p.12). As a result, exploring generational changes in health behaviors could not only help gain a deeper understanding of ethnic health disadvantages but could also shed light on public policy in terms of providing culturally diverse health care services and promoting healthy lifestyles. Thus, this article contributes to previous research by exploring whether British ethnic minorities have undergone a generational transition in health behaviors and whether such transitions, if any, can be explained by their SES and degree of ethnic identity.

In terms of smoking behavior, we find that second-generation Indians, Pakistanis, and Black Caribbeans have a significantly higher probability of smoking than their first-generation counterparts. This result lends some support to classical straight-line assimilation theory and is consistent with previous studies from the U.S. [5,6]. The relatively high probability of smoking may imply potential health risks for the second-generation of these groups, which could exacerbate ethnic inequalities in health and may warrant policy intervention. Importantly, we find that the generational differences in smoking behavior are not explained by generational differences in SES and ethnic identity, highlighting that other unobserved characteristics may play a role in explaining the generational transition in smoking behavior. For Bangladeshis and Black Africans, there is little evidence for generational changes in smoking behavior. One possible explanation could be that both groups may be well protected by their ethnic communities from adopting unhealthy behavior or lifestyles in the host society [9].

In terms of alcohol consumption, we find strong evidence of generational changes for all ethnic groups with the second-generation being significantly more likely to consume alcohol than the first-generation. This result is consistent with previous studies from the U.S. [5,6] and suggests that high levels of alcohol consumption among the second-generation may be a potential reason for ethnic minorities’ generational deterioration of health, highlighting the need for policy intervention. Moreover, these generational differences are partly explained by second-generation ethnic minorities’ higher SES and weakened ethnic identity. Although ethnic minorities’ upward social mobility is often associated with the improvement of health, the process of social mobility could also mean that ethnic minorities have more interactions with White British and higher levels of exposure to White British culture and lifestyles and gradually attach less importance to their home country’s traditions regarding alcohol drinking [4].

In terms of dietary style (i.e., the number of fruits and vegetables eaten per day), we find that both first- and second-generation ethnic minorities are significantly less likely to eat fruits and vegetables than White British to a similar degree, and there are no significant generational differences. One exception is Indians, but the size of the generational difference for Indians is very small. Overall, this pattern is partly because dietary habits are primarily socialized within families, whereas the formation of smoking and drinking behaviors may also depend on socialization in other social settings such as schools, neighborhoods, and workplaces. Nevertheless, the lower levels of fruit and vegetable consumption among both first- and second-generation ethnic minorities compared to White British may be an essential reason for the poor health of ethnic minorities and warrant health policy intervention.

### Limitations and Future Research Directions

Despite several contributions to previous literature, there are several limitations in this study that could be the focus of future research. First, due to data limitation, this article primarily used migration generation and ethnic identity to measure acculturation, but such a measure is incomplete and may only capture a single dimension of acculturation. Future research could profitably use more comprehensive measures of ethnic acculturation such as length of time stayed in the host society, the language used at home, inter-ethnic social networks, and neighborhood ethnic composition. Second, there are substantial remaining unexplained generational differences in smoking behavior and alcohol consumption net of demographic characteristics, SES, and ethnic identity, highlighting the complexity of generational transition in health behaviors. Future research could explore whether such generational differences could be explained by other ethnic minorities’ characteristics that were not included in this research. Finally, because all ethnic minorities analyzed in this study came from previous British colonial countries, they might have been influenced by British culture to a varying degree before migration, depending on the duration of colonization. Future research exploring the mechanisms behind the generational transition in health behaviors may need to distinguish between the effects of sending and host societies.

## 5. Conclusions

By exploring ethnic generational differences in smoking behavior, frequency of alcohol consumption, and dietary style, this article makes significant contributions to previous literature in terms of facilitating a better understanding of ethnic minorities’ generational transitions in health behaviors, highlighting the need for future policy interventions to target certain second-generation ethnic minorities who have adopted certain host society unhealthy lifestyles. In addition, this article also raises important questions for future research by emphasizing the complex mechanisms of generational transition in health behaviors.

## Figures and Tables

**Table 1:** Descriptive statistics by ethnicity. M = Means, % = Proportion.

	White British	Indian	Pakistani	Bangladeshi	Black Caribbean	Black African	F/χ^2^ *p*-Value
**Gender (%)**							
Male	44.95	52.31	45.94	47.21	37.58	42.68	<0.001
**Age (M)**	48.69	41.94	37.22	35.66	46.63	37.19	<0.001
Standard deviations	18.76	16.01	15.25	14.36	18.04	14.15	
**Marital status (%)**	33.46	26.04	30.52	37.3	52.69	45.14	<0.001
Single/never married	33.46	26.04	30.52	37.3	52.69	45.14	
Married/cohabited	52.41	67.75	61.56	55.68	28.8	41.08	
Separated/divorced/widowed	14.13	6.21	7.92	7.03	18.51	13.79	
**Urban/rural area (%)**							
Urban	71.29	98.11	99.38	98.92	99.34	98.58	<0.001
**Occupational class (%)**							<0.001
Inactive	34.76	25.98	44.06	37.66	30.88	29.18	
Unemployed	4.86	7.51	8.44	10.81	12.18	10.10	
Working class	20.62	21.12	18.44	23.6	20.21	27.48	
Intermediate class	14.22	14.14	13.54	13.33	15.68	10.2	
Salariat class	25.54	31.24	15.52	14.59	21.06	23.04	
**Equivalised household income £ (M)**	2029.76	2072.83	1276.59	1387.69	1708.81	1664.88	<0.001
Standard deviations	1286.05	1436.54	757.69	839.30	1040.02	996.50	
**Smoke (%)**							
Yes	20.05	10.36	11.67	15.32	23.14	8.31	<0.001
**Drinking frequency (M)**	4.51	2.89	1.42	1.47	3.56	2.73	<0.001
Standard deviations	1.85	1.82	1.12	1.09	1.87	1.76	
**Number of servings of fruits or vegetables (M)**	3.36	3.00	2.62	2.60	3.03	2.82	<0.001
Standard deviations	1.57	1.43	1.36	1.34	1.58	1.41	
**Importance of ethnic background (%)**							<0.001
Very important	16.9	44.56	57.71	53.87	61.19	62.32	
Fairly important	25.64	31.54	29.38	28.29	21.44	21.72	
Not very important	30.96	17.16	9.17	11.89	11.61	11.99	
Not important	26.51	6.75	3.75	5.95	5.76	3.97	
*N*	59,141	1690	960	555	1059	1059	

**Table 2:** Descriptive statistics by generation within ethnic minority groups. M = Means, % = Proportion.

	Indian		Pakistani		Bangladeshi		Black Caribbean		Black African		F/χ^2^
	1st gen.	2nd gen.	1st gen.	2nd gen.	1st gen.	2nd gen.	1st gen.	2nd gen.	1st gen.	2nd gen.	*p*-Value
**Gender (%)**											
Male	55.68	47.59	51.66	41.45	54.26	41.08	41.43	34.75	43.18	40.87	<0.001
**Age (M)**	47.16	32.42	44.50	29.39	40.79	26.49	56.97	38.18	39.27	29.29	<0.001
Standard deviations	14.28	9.35	12.41	13.23	15.26	9.47	16.04	11.70	14.35	11.82	
**Marital status (%)**											<0.001
Single/never married	10.34	48.01	9.48	47.03	5.81	64.65	32.52	67.54	37.03	74.35	
Married/cohabited	82.25	47.44	80.33	46.84	82.95	31.99	38.75	21.48	47.53	17.83	
Separated/divorced/widowed	7.4	4.55	10.19	6.13	11.24	3.37	28.73	10.98	15.44	7.83	
**Urban/rural area (%)**											<0.05
Urban	98.28	97.87	99.76	99.07	100	97.98	99.11	99.51	98.43	99.13	
**Occupational class (%)**											<0.001
Inactive	29.82	20.6	47.16	41.64	37.21	38.05	46.1	19.67	27.38	35.65	
Unemployed	6.49	8.95	7.82	8.92	10.85	10.77	7.13	15.9	10.01	10.43	
Working class	24.24	16.76	18.25	18.59	25.97	21.55	21.16	19.51	29.67	19.57	
Intermediate class	12.17	16.9	12.32	14.5	17.44	9.76	10.47	19.51	10.37	9.57	
Salariat class	27.28	36.79	14.45	16.36	8.53	19.87	15.14	25.41	22.56	24.78	
**Equivalised household income £ (M)**	2054.73	2098.39	1240.27	1304.05	1321.04	1444.95	1595.48	1791.41	1622.04	1819.74	<0.001
Standard deviations	1468.05	1705.35	738.92	779.52	1103.25	947.03	984.18	1074.32	978.53	1139.04	
**Smoke (%)**											
Yes	8.11	13.49	10.43	12.64	14.34	16.16	14.48	29.51	6.51	14.78	<0.001
**Drinking frequency (M)**	1.34	1.60	1.27	1.50	2.62	3.28	3.27	4.01	2.61	2.98	<0.001
Standard deviations	0.98	1.03	0.99	1.02	1.45	1.67	1.87	2.01	1.02	1.38	
**Number of servings of fruits or vegetables (M)**	3.12	2.83	2.66	2.58	2.65	2.54	3.19	2.90	2.84	2.74	
Standard deviations	1.26	1.26	1.31	1.29	1.58	1.41	1.49	1.50	1.39	1.36	
**Importance of ethnic background (%)**											<0.001
Very important	47.77	40.06	60.19	55.76	66.28	43.10	64.37	58.85	64.54	54.35	
Fairly important	27.99	36.51	28.67	29.93	23.26	32.66	18.49	23.61	19.18	30.87	
Not very important	16.84	17.61	6.87	10.97	7.75	15.49	10.69	12.30	11.82	12.61	
Not important	7.40	5.82	4.27	3.35	2.71	8.75	6.46	5.25	4.46	2.17	
*N*	986	704	422	538	258	297	450	610	829	230	

Note: 1st gen. refers to the first generation, and 2nd gen. refers to the second generation.

**Table 3:** Logistic regression models predicting ethnic minority generational differences in the probability of being current smoker (vs. non-current smoker).

	Indians	Pakistanis	Bangladeshis	Black Caribbeans	Black Africans
**Model 1 (adjusting for demographics)**					
Ethnicity (ref. = White British, *N* = 59,189)					
First-generation	−0.14 ***	−0.09 ***	0.01	−0.07 ***	−0.16 ***
	(0.01)	(0.02)	(0.03)	(0.02)	(0.01)
Second-generation	−0.12 ***	−0.10 ***	−0.08 **	0.05 *	−0.12 ***
	(0.01)	(0.02)	(0.03)	(0.02)	(0.02)
**Model 2 (adjusting for demographics)**					
Generation (ref. = First-generation)					
Second-generation	0.10 ***	0.08 **	−0.04	0.21 ***	0.04
	(0.02)	(0.03)	(0.06)	(0.03)	(0.02)
**Model 3 (adjusting for demographics and SES)**					
Generation (ref. = First-generation)					
Second-generation	0.10 ***	0.10 ***	−0.01	0.20 ***	0.04
	(0.02)	(0.03)	(0.05)	(0.04)	(0.03)
**Model 4 (adjusting for demographics, SES and ethnic identity)**					
Generation (ref. = First-generation)					
Second-generation	0.10 ***	0.09 **	0.00	0.20 ***	0.04
	(0.02)	(0.03)	(0.06)	(0.04)	(0.02)
*N*	1690	960	555	1060	1059

Note: Models report average marginal effects and robust standard errors in parentheses, *** *p* < 0.001, ** *p* < 0.01, * *p* < 0.05. SES: socioeconomic status.

**Table 4:** Linear regression models predicting ethnic minority generational differences in the frequency of alcohol consumption.

	Indians	Pakistanis	Bangladeshis	Black Caribbeans	Black Africans
**Model 1 (adjusting for demographics)**					
Ethnicity (ref. = White British, *N* = 59,189)					
First-generation	−1.95 ***	−3.32 ***	−3.28 ***	−1.10 ***	−1.87 ***
	(0.07)	(0.06)	(0.10)	(0.10)	(0.08)
Second-generation	−1.32 ***	−2.98 ***	−2.65 ***	−0.45 ***	−1.48 ***
	(0.09)	(0.07)	(0.15)	(0.10)	(0.14)
**Model 2 (adjusting for demographics)**					
Generation (ref. = First-generation)					
Second-generation	0.83 ***	0.29 **	0.75 **	0.82 ***	0.33 *
	(0.13)	(0.11)	(0.25)	(0.16)	(0.16)
**Model 3 (adjusting for demographics and SES)**					
Generation (ref. = First-generation)					
Second-generation	0.75 ***	0.29 *	0.67 **	0.73 ***	0.27
	(0.13)	(0.12)	(0.26)	(0.16)	(0.16)
**Model 4 (adjusting for demographics, SES, and ethnic identity)**					
Generation (ref. = First-generation)					
Second-generation	0.73 ***	0.30 *	0.57 *	0.74 ***	0.30
	(0.13)	(0.12)	(0.26)	(0.16)	(0.16)
*N*	1690	960	555	1060	1059

Note: Models report coefficients and robust standard errors in parentheses, *** *p* < 0.001, ** *p* < 0.01, * *p* < 0.05.

**Table 5:** Poisson regression models predicting ethnic minority generational differences in the number of servings of fruits or vegetables eaten per day.

	Indians	Pakistanis	Bangladeshis	Black Caribbeans	Black Africans
**Model 1 (adjusting for demographics**)					
Ethnicity (ref. = White British, *N* = 59,189)					
First-generation	−0.07 ***	−0.23 ***	−0.18 ***	−0.06 *	−0.14 ***
	(0.02)	(0.02)	(0.03)	(0.03)	(0.02)
Second-generation	−0.13 ***	−0.21 ***	−0.11 *	−0.10 ***	−0.17 ***
	(0.02)	(0.03)	(0.04)	(0.03)	(0.04)
**Model 2 (adjusting for demographics)**					
Generation (ref. = First-generation)					
Second-generation	−0.07 *	0.04	0.08	−0.02	−0.02
	(0.03)	(0.04)	(0.06)	(0.05)	(0.05)
**Model 3 (adjusting for demographics and SES)**					
Generation (ref. = First-generation)					
Second-generation	−0.08 *	0.05	0.05	−0.04	−0.03
	(0.03)	(0.04)	(0.06)	(0.05)	(0.05)
**Model 4 (adjusting for demographics, SES and ethnic identity)**					
Generation (ref. = First-generation)					
Second-generation	−0.08 *	0.04	0.05	−0.03	−0.04
	(0.03)	(0.04)	(0.06)	(0.05)	(0.05)
*N*	1690	960	555	1060	1059

Note: Models report coefficients and robust standard errors in parentheses, *** *p* < 0.001, ** *p* < 0.01, * *p* < 0.05.

## Appendix A

**Table A1:** Logistic regression models predicting ethnic minority generational differences in the probability of being an ever smoker (vs. never smoker).

	Indians	Pakistanis	Bangladeshis	Black Caribbeans	Black Africans
**Model 1 (adjusting for demographics and SES)**					
Ethnicity (ref. = White British, *N* = 59,189)					
First-generation	−0.42 ***	−0.36 ***	−0.25 ***	−0.29 ***	−0.38 ***
	(0.01)	(0.02)	(0.03)	(0.02)	(0.02)
Second-generation	−0.24 ***	−0.28 ***	−0.24 ***	0.03	−0.22 ***
	(0.02)	(0.03)	(0.04)	(0.03)	(0.04)
**Model 2 (adjusting for demographics)**					
Generation (ref. = First-generation)					
Second-generation	0.20 ***	0.14 ***	0.00	0.28 ***	0.14 **
	(0.03)	(0.04)	(0.06)	(0.04)	(0.05)
**Model 3 (adjusting for demographics and SES)**					
Generation (ref. = First-generation)					
Second-generation	0.20 ***	0.13 **	0.01	0.28 ***	0.12 *
	(0.03)	(0.04)	(0.05)	(0.04)	(0.05)
**Model 4 (adjusting for demographics, SES and ethnic identity)**					
Generation (ref. = First-generation)					
Second-generation	0.20 ***	0.13 **	0.03	0.28 ***	0.12 *
	(0.03)	(0.04)	(0.06)	(0.04)	(0.05)
*N*	1690	960	555	1060	1059

Note: Models report average marginal effects and robust standard errors in parentheses, *** *p* < 0.001, ** *p* < 0.01, * *p* < 0.05.

**Table A2:** Logistic regression models predicting ethnic minority generational differences in the probability of having five or more servings of fruits or vegetables per day.

	Indians	Pakistanis	Bangladeshis	Black Caribbeans	Black Africans
**Model 1 (adjusting for demographics)**					
Ethnicity (ref. = White British, *N* = 59,189)					
First-generation	−0.03 *	−0.13 ***	−0.06	−0.01	−0.08 ***
	(0.01)	(0.02)	(0.04)	(0.02)	(0.02)
Second-generation	−0.08 ***	−0.08 ***	−0.08 *	−0.05 *	−0.09 **
	(0.02)	(0.02)	(0.04)	(0.02)	(0.03)
**Model 2 (adjusting for demographics)**					
Generation (ref. = First-generation)					
Second-generation	−0.04	0.03	−0.01	−0.05	0.01
	(0.02)	(0.02)	(0.05)	(0.04)	(0.03)
**Model 3 (adjusting for demographics and SES)**					
Generation (ref. = First-generation)					
Second-generation	−0.05 *	0.03	−0.02	−0.06	0.00
	(0.02)	(0.02)	(0.04)	(0.03)	(0.03)
**Model 4 (adjusting for demographics, SES and ethnic identity)**					
Generation (ref. = First-generation)					
Second-generation	−0.05 *	0.03	−0.04	−0.05	0.00
	(0.02)	(0.03)	(0.04)	(0.03)	(0.03)
*N*	1690	960	555	1060	1059

Note: Models report average marginal effects and robust standard errors in parentheses, *** *p* < 0.001, ** *p* < 0.01, * *p* < 0.05.
